# Predictors of Developmental and Adaptive Behaviour Outcomes in Response to Early Intensive Behavioural Intervention and the Early Start Denver Model

**DOI:** 10.1007/s10803-023-05993-w

**Published:** 2023-05-12

**Authors:** Catherine Bent, Susan Glencross, Karen McKinnon, Kristelle Hudry, Cheryl Dissanayake, The Victorian ASELCC Team, Giacomo Vivanti

**Affiliations:** 1 Department of Psychology, Counselling and Therapy, School of Psychology and Public Health, La Trobe University, Melbourne, Australia; 2 Autism Partnership, Melbourne, Australia; 3 Olga Tennison Autism Research Centre, School of Psychology and Public Health, La Trobe University, Melbourne, Australia; 4 A.J. Drexel Autism Institute, Drexel University, Philadelphia, USA

**Keywords:** Autism, Early start denver model, Applied behaviour analysis, Predictors, Prognostic indicators, Early intervention, Early intensive behavioural intervention, Naturalistic developmental behavioural intervention

## Abstract

Many autistic children require support to meet their learning needs. Given the heterogeneity within the autism spectrum it is plausible that different types of support might be better suited to different children. However, knowledge on what interventions work best for which children is limited. We examined the outcomes of autistic preschool-aged children receiving one of two community early intervention approaches. Our main objective was to understand which baseline child characteristics might be associated with the degree of individual response to intervention—whether prognostically (i.e., irrespective of intervention received) or predictively (i.e., specifically in the context of one or other EI approach). Participants comprised two matched groups of preschool-aged autistic children receiving either Group-based Early Start Denver Model (G-ESDM; n = 42) delivered in a 1:3–4 staff:child ratio or an Early Intensive Behavioural Intervention (EIBI; n = 40) delivered in combination of 1:1 and 1:2 staff:child ratio. Over an approximate one-year follow-up period, children in both groups made significant gains in Developmental Quotient (DQ) scores, and trend-level gains in adaptive behaviour composite scores. Higher attention to a playful adult measured via an eye-tracking task was prognostically indicative of better verbal DQ and adaptive behaviour outcomes for the cohort overall. Moderation analyses indicated a single predictive effect—of pre-program sustained attention for subsequent NVDQ outcomes specific to those children receiving G-ESDM. These findings suggest that fine-grained measures of learning skills offer promise towards the selection and tailoring of intervention approaches to meet individual children’s learning needs.

**Supplementary Information:**

The online version contains supplementary material available at 10.1007/s10803-023-05993-w.

Autism spectrum disorder (ASD; hereafter autism) is characterised by social communication differences and the presence of strong and/or specific behaviours and interests (American Psychiatric Association, 2013). It is a highly heterogeneous neurodevelopmental condition, with substantial variation in the type and level of autism characteristics and the impact of these on how individuals learn and interact within their environment. Intervention delivered early in life has the potential to support the wellbeing, development and quality of life of autistic children. Support needs in the autistic population are highly variable, and approaches differ in their intensity, format, and application, as well as in the theoretical mechanisms proposed to drive outcomes. However, the limited research informing the tailored selection of approaches for individual children means that decisions are often guided by availability, personal recommendations, and beliefs about autism (Miller et al., 2012; Mire et al., 2017; Trembath et al., 2021) rather than evidence around a particular approaches alignment with a child’s skills and needs.

Research investigating early intervention outcomes has traditionally focused on understanding group-level effects; the extent to which a particular intervention works *on average* to support developmental outcomes. This approach fails to consider individual differences in intervention response, despite appreciation of substantial variability in the course of autism in early childhood and across the lifespan (Trembath & Vivanti, 2014). Understanding individual differences and identifying those characteristics associated with better or poorer intervention response is critical, as different approaches might be particularly well indicated or even contra-indicated for particular individuals.

Two increasingly adopted intervention approaches are Early Intensive Behavioural Intervention (EIBI; Leaf & McEachin, 1999; Lovaas, 1987), and the Early Start Denver Model (ESDM; Dawson et al., 2010; Rogers et al., 2012a, 2012b), a naturalistic developmental behavioural intervention (NDBI; Schreibman et al., 2015). Both are based on behavioural principles of applied behaviour analysis (ABA; Vivanti & Stahmer, 2021) and outcome studies suggest each is efficacious in improving young autistic children’s cognition, adaptive behaviour, and social-communication skills (Dawson et al., 2010; Howard et al., 2014; Rogers et al., 2012a).

In the ESDM, behavioural strategies are solely employed within a naturalistic social context, and in line with developmental principles (i.e., incorporating the child’s interests and choices, adult sensitivity and responsiveness, child affect and arousal, and targeting developmentally appropriate sequences of skills/behaviours). The ESDM relies on social engagement to motivate children to participate in and learn from joint routines (Rogers & Geraldine, 2010; Vivanti et al., 2017a). EIBI also adopts a developmental framework, however, implementation can range from highly structured learning environments and adult-led activities, through to naturalistic and incidental teaching, depending on the needs of the child. Some EIBI approaches are highly protocol-driven while more progressive approaches include a range of flexible and responsive behavioural techniques (Leaf et al., 2016). An initial focus of EIBI often includes “learning how to learn”, where the skills required to learn in different developmental domains are explicitly taught and generalised attention to both social and non-social activities is promoted.

## Individual Determinants of Early Intervention Response

Studies investigating the individual characteristics associated with children’s early intervention outcomes have reported better response when children have commenced intervention at a younger age (e.g., Clark et al., 2018; Flanagan et al., 2012; Frazier et al., 2021; Vivanti et al., 2019), with higher level of communication skills (e.g., Laister et al., 2021; Sievers et al., 2018), fewer core autism features (e.g., Eapen & Crncec, 2016; Sievers et al., 2018), and higher cognitive abilities and adaptive behaviours (e.g., Hudry et al., 2018; Sinai-Gavrilov et al., 2020; Tiura et al., 2017). However, recent systematic reviews and meta-analyses examining the role of child age and baseline cognitive ability, and also the amount of intervention received, suggest findings across studies remain inconsistent and advocate the need for further research with controlled designs to better understand how pre-program characteristics are associated with intervention-related outcomes (e.g., Vivanti et al., 2014; Whitehouse et al., 2020).

Most studies to date that have examined the outcomes of autistic children receiving a particular early intervention have employed single group pre-test post-test designs, or comparison to a non-specified ‘treatment as usual’ group. Few studies have investigated children’s outcomes as a result of accessing different types of intervention. One notable exception is a recent trial comparing the effects of ESDM and EIBI at two levels of intensity (15 vs. 25 h per week) over a 12-month period (Rogers et al., 2021). Results indicated significant gains in non-verbal abilities, and expressive and receptive language as well as reduced autism behaviours in response to both approaches. Baseline core autism features and cognitive ability were investigated as potential moderators, with no evidence of differential outcomes by intervention approach. This recent study provides a strong test of differential intervention effects and evidence that both EIBI and ESDM provided at 15–25 h per week promote positive developmental outcomes for young autistic children. However, it leaves open the question ‘What works for whom?’ with no evidence to date informing whether particular child characteristics might differentiate response to one vs. another early autism intervention approach.

Discrete skills, proximally related to program goals, may have better prognostic value than broad, composite measures of developmental level or core autism features (Vivanti et al., 2020), and could offer a potential ‘precision-medicine’ approach to intervention selection and delivery. However, very few studies have examined the potential predictive utility of discrete measures. We have previously developed eye-tracking measures of discrete characteristics hypothesised to support the social-mediated learning (Vivanti et al., 2016, 2017a) and preliminary data suggest association with higher developmental gains (Vivanti et al., 2013) . Independently, Smith et al. (2015) have examined predictors of intervention response among 71 children receiving EIBI and found baseline measures of social engagement (social attention, joint attention and imitation) to predict developmental and adaptive behaviour outcomes. However, neither their nor our own past work has included a comparison group precluding determination of associations reflecting specific predictors of outcomes from a given intervention approach vs. more general indicators of favourable prognosis.

## The Current Study

We sought to identify whether discrete characteristics might predict differential child response to one or other of two intervention approaches for autistic preschool-aged children available within the same community: EIBI and ESDM. We anticipated significant group mean-level gains in developmental skills and adaptive behaviours following one year of intervention for children receiving either program. Further, we hypothesised that baseline characteristics such as core autism features, developmental level, domain-general attention skills (e.g., sustained attention) and other specific social skills (i.e., social interest, joint attention, imitation) would evidence prognostic value (i.e., association with intervention-related outcomes). Based on the theoretical premises of EIBI and ESDM approaches, we expected that the predictive value of baseline child sustained attention and social indicators for subsequent outcomes would be moderated by intervention approach. Specifically, that social attention might be more strongly predictive of outcomes for preschool-aged children receiving ESDM, given the fundamental role of social engagement in ESDM, while sustained attention might be more strongly predictive of outcomes for those receiving EIBI, given the importance of domain-general attention skills.

## Method

### Design

The study utilised a pretest–posttest design with convenience sampling of 89 preschool-aged children recruited from two independent community-based intervention services, with approval by the La Trobe University Human Ethics Committee (Ref 14-007). Parents/caregivers provided signed informed consent for their child’s participation in this research, after they had made the independent decision to enroll their child in a particular service. Families accessed the services through alternate pathways (e.g., self-referral, word-of-mouth, or recommendation from local health professionals/services). Exclusion criteria included uncorrected visual impairment or a diagnosed co-occurring condition known to affect neurological and developmental abilities. Standardised measures of child developmental level and behaviour were completed at program intake (hereafter, Time 1) alongside a novel battery of eye-tracking tasks designed to characterise early learning skills. Standardised measures were re-administered after approximately one year of intervention (Time 2; *M*(*SD*) = 10.87(1.57) months).

### Intervention Contexts

*Group Early Start Denver Model (G-ESDM).* Forty-six children were enrolled in a G-ESDM (Vivanti et al., 2017b) program at  the Victorian Autism Specific Early Learning and Care Centre (ASELCC) at La Trobe University in Melbourne, Australia. The G-ESDM is a manualised group-based adaption of the ESDM intended for classroom environments and small group settings (Zitter et al., 2022). Children received approximately 15 h of intervention across 3 days per week for one year (February to December). G-ESDM was delivered by trained therapy assistants alongside at least one ESDM-certified therapist, at a 1:3–4 staff:child ratio. An on-site team of ESDM-certified allied health/education professionals provided additional specialist support as needed, within the classroom setting. An ESDM Curriculum Checklist assessment (Dawson et al., 2010) was conducted by an ESDM certified therapist at the beginning of the enrolment year, and quarterly thereafter, to inform each child’s goals. Goals were developed in consultation with parents and target multiple developmental domains including communication, play, daily living skills, social engagement, and cognition. Naturalistic developmental behavioural learning strategies are implemented during daily routines and target children’s individual learning goals within group-based cooperative activities (Vivanti et al., 2017b). For example, verbal and non-verbal communication, as well as cognitive and adaptive skills like requesting a turn during a group activity, indicating a preference between different options, sharing attention and interest with peers and adults, and engaging in hygiene and safety routines are learned and practiced in the context of activities and materials that resemble those used in typical settings. The focus on shared naturalistic and playful routines (e.g., ‘arts and craft’, ‘music and movement’ etc.) as the context for embedding targeted teaching is designed to be in contrast with artificial “therapy room” situations, offering the potential to embed learning within culturally relevant shared experiences, naturally occurring contingencies, and situations in which the skills being taught are meant to be used (Vivanti et al., 2017b).

The ESDM Fidelity Scale (Dawson et al., 2010) and the G-ESDM Group Activity Fidelity Coding Tool (Vivanti et al., 2017b) were used during initial training and ongoing coaching to monitor adherence to the ESDM manualized practices across teaching strategies and classroom group-based routines. An ESDM-certified allied health/education professional conducted formal observations at least twice-yearly assessing individual and group level fidelity. Deviations from fidelity (operationalized as scores below 80% in the fidelity checklists) were addressed via performance feedback and focused ‘booster’ training sessions.

### Early Intensive Behavioural Intervention (EIBI)

Forty-three children were enrolled in an EIBI program across three sites at Autism Partnerships (Melbourne, Geelong, and Sydney), receiving 19.5–27 h per week of intervention at a 1:1–2 staff:child ratio (including 1:1 delivery from 0 to 21 h perweek, with 38/43 children receiving this full amount). Intervention was delivered by trained behaviour therapists, under supervision of a behaviour consultant with a Masters in Applied Behaviour Analysis or Psychology/other relevant degree and ≥ 5 years practical experience in ABA, including an internship program at Autism Partnerships. Children’s goals were developed in consultation with parents and informed by a manualised curriculum (e.g., Leaf & McEachin, 1999) and addressed a comprehensive range of developmental areas (e.g., language, communication, social engagement, play, behaviour regulation).

Behaviour therapists created learning opportunities, which were individualised to the child on a continuum from structured to naturalistic. Whilst some learning opportunities were embedded into daily routines (e.g., morning arrival routine, mealtimes, small group lessons, social exchanges), others were contrived to allow the child to experience repeated opportunities to practice the target skill. Across the day, each child spent time working on their own individual goals, within the same teaching space as other children, or in other learning environments (e.g., preschool, home). Some of these goals were implemented via 1:1 teaching format and others were delivered in small groups. Behaviour therapists used in-the-moment assessment to determine what set of learning conditions (e.g., 1:1/ small group, structured/ naturalistic) a child needed at any point in time (Leaf et al, 2016). Direct intervention occurred in a centre-based autism-specific learning environment, at the child’s preschool setting or in the home and community, dependent on the child’s needs across time. Family participation was integrated into the intervention service in various formats, such as goal setting and review meetings, one-on-one meetings with the behaviour consultant, observations and support in the home and collaboration and participation in whole team meetings.

Autism Partnerships training and quality assurance procedures included procedural fidelity checks to ensure quality of intervention delivery. Initial training to fidelity was achieved via a competency assessment based on progressive discrete trial teaching techniques (Leaf et al., 2016; Milne et al., 2022). Continuous monitoring and feedback on fidelity of implementation was provided through direct observation and ongoing scoring on the competency assessment by the behaviour consultants and senior clinicians who supervised the behaviour therapists.

### Participant Characteristics and Matching

Most of the cohort of 89 preschool-aged children were from two-parent households (84.6%) with parents educated at tertiary-level (88.4% mothers, 76.4% fathers). Parents predominately self-identified as being of Asian (39% mothers, 41.4% fathers), Australian (36.4% Mothers, 24.3% fathers), or European (18.2% mothers, 25.7% fathers) background. The groups were matched on child sex and Time 1 core autism features. The EIBI group had significantly lower Time 1 DQ than the G-ESDM group (see Table 1) so we excluded data for seven participants—four G-ESDM participants with high- and three EIBI participants with low Time 1 DQ—thereby achieving matching on DQ, and also adaptive behaviour measures for the retained sample of 82 participants. G-ESDM participants were significantly younger than their EIBI counterparts, so Time 1 age was included as a statistical covariate.

**Table 1:** Baseline sample characteristics

	Recruited sample			Matched sample retained for analysis		
	ESDM (N = 46)	EIBI (N = 43)	*p*	ESDM (N = 42)	EIBI (N = 40)	*p*
Sex (Male N,%)	33 (71%)	37 (86%)	.100	31 (73%)	34 (85%)	.279
Age at intake	32.20 (7.83)	42.56 (9.35)	< .001	31.64 (7.42)	43.03 (9.10)	< .001
ADOS-SA	13.06 (4.06)	12.60 (3.65)	.572	13.57 (3.78)	12.50 (3.68)	.195
ADOS-RBB	4.96 (1.93)	4.92 (1.63)	.934	5.07 (1.93)	4.86 (1.61)	.609
NonVerbal-DQ	71.10 (19.87)	58.70 (19.87)	.008	66.95 (18.14)	61.32 (18.15)	.164
Verbal-DQ	56.50 (29.14)	42.68 (24.03)	.017	51.87 (25.79)	44.54 (23.77)	.185
Total-DQ	63.14 (25.53)	49.57 (18.91)	.004	59.07 (20.83)	51.69 (17.81)	.089
VABS-ABC	71.19 (8.14)	67.60 (10.60)	.082	70.57 (8.08)	68.40 (10.71)	.302

Statistics are mean (standard deviation), unless otherwise specified, *p* values derived from chi square and simple t-tests

*ADOS*:Autism diagnostic observation schedule, *SA*:Social affect total score, *RRB*:Restricted repetitive behaviour total score, *DQ*:Developmental quotient score from mullen scales early learning, *VABS-ABC*:Vineland adaptive behaviour scales adaptive behaviour composite score

### Standardised Measures

Assessments were conducted at the centre where children were enrolled, by a trained psychologist or researcher. The Autism Diagnostic Observation Schedule – 2nd Edition (ADOS-2; Lord et al., 2012) was administered at Time 1 to confirm autism diagnosis, with Social Affect (SA) and Restricted and Repetitive Behaviour (RRB) algorithm total scores retained as measures of core autism features. The primary outcome measures used to appraise intervention response, taken at both Time 1 and Time 2, were the Mullen Scales of Early Learning (MSEL; Mullen, 1995) and the Vineland Adaptive Behaviour Scales (2nd and 3rd Editions; (VABS; Sparrow et al., 2005, 2016). The MSEL is a direct standardised assessment of early developmental abilities yielding four domain age-equivalence (AE) scores (receptive language, expressive language, visual reception, and fine motor) and summary Developmental Quotients (overall DQ; and verbal and non-verbal V/NVDQ). The Vineland Adaptive Behaviour Scales (2nd and 3rd Editions; (VABS; Sparrow et al., 2005, 2016) is a parent-report measure of adaptive functioning, yielding domain-level scores across Communication, Daily Living, Socialisation and Motor skills, and an overall Adaptive Behaviour Composite (ABC) Standard Score (SS).

### Eye-Tracking Measures

A battery of eye-tracking tasks offering fine-grained evaluation of the skills supporting learning for all children (Vivanti et al., 2016, 2017a) was administered. Stimuli were presented via table-top computer monitor with participants seated approximately 60 cm away, and eye movements recorded via Tobii X2-60 eye-tracker, at 60 Hz sampling rate, with areas of interest defined using Tobi Studio software. Following an initial five-point calibration procedure, tasks were presented in one of two fixed random orders, counterbalanced across participants. A central fixation cross appeared for one second before each new task, and ‘filler’ stimuli between tasks served to maintain children’s attention.

*Sustained Attention.* Participants viewed a 5-min children’s animation ‘Spot the Dog’. Total duration of gaze to the animation was used as an index of sustained attention (Graziano et al., 2011; Vivanti et al., 2017a). Concurrent validity is supported by the moderate positive correlations evident between sustained attention and measures of duration of attention to other eye tracking stimuli (r = 0.385** to 0.680**, see supplementary materials).

*Preferential Social Attention.* Preferential attention to social stimuli was measured following (Vivanti et al., 2017a) procedure, with average duration of attention to social and non-social targets across eight stimuli (5 images; 3 short videos) recorded. Correlations between preferential social attention and related measures of joint attention and attention to a playful adult (r = 0.392 to 0.508), and standardised measures of social and communication skills (r = 0.336 to 0.398) support the concurrent validity of this measure (see supplementary materials).

*Response to Joint Attention.* Participants’ ability to follow another’s gaze was measured using a joint attention paradigm (Vivanti et al., 2017a) in which participants viewed a video where an actor established direct gaze, then looked away toward one of two objects. Across six trials (11 s each), the proportion of trials in which a child first looked to the target object, and total duration of attention to the target were used as joint attention indices. Concurrent validity is supported by strong positive correlations between the two indicators of response to joint attention (r = 0.680) and small to moderate associations with standardised measures of social and communication skills (r = 0.156 to 0.499, see supplementary materials).

*Attention to Playful Adult.* Vivanti et al.’s (2016) eye-tracking paradigm was used to measure attention to and imitation of a playful adult. Across eight trials and two conditions (playful vs. neutral), children viewed a 10-s video where the adult demonstrated a simple non-functional action involving one of eight available objects. Participants were then presented with the identical object set and (with no explicit instruction given) their spontaneous behaviour was filmed for later coding by researchers kept blind to the study aims, assessment time point, and other child characteristics. Imitation was scored against the following criteria: participant imitated the demonstrated action (3 points), imitated after a delay (i.e., in subsequent trial; 2 points), touched the target object without imitating (1 point), and did not touch target object (0 points). Key metrics of interest here were total duration of attention to the playful actor, and imitation score. Concurrent validity is supported by expected correlations between attention to a playful adult and other measures of preferential social attention and joint attention, as well as with standard measures of social and communication skills (r = 0.245 to 0.386, see supplementary material). Imitation performance was also associated with attention to a playful adult within the same paradigm (r = 0.342).

### Data Preparation

Missing Values Analysis revealed 4.8% missing data on key standardized measures. As Little’s MCAR test indicated these were missing completely at random, *χ*^*2*^(49) = 49.13, *p* = 0.468, data were imputed via Expectation Maximization. Data comparison before and after imputation is presented in supplementary materials. Several positively-skewed MSEL domain AEs and eye-tracking metrics were corrected using Square-Root Transformation for parametric analysis. Univariate outliers were trimmed to the next most extreme value ± 1, and one identified multivariate outlier was removed from the regression analysis predicting Time 2 VABS-ABC.

### Statistical Analyses

The hypothesis that children in both groups would make significant developmental and adaptive behaviour gains was examined using 2 Group (EIBI, ESDM) × 2 Timepoint ANCOVAs. To examine prognostic indicators of intervention outcome, hierarchical regressions were conducted to determine which Time 1 characterisation measures accounted for most variance in Time 2 MSEL-DQs and VABS-ABC, across both groups. Control measures entered at Step 1 included child sex, ADOS-SA and RRB scores, and Time 1 level of the given outcome measure, followed by potential predictors (measures significantly correlated with the specified outcome) at Step 2. Partial eta squared was used as a measure of effect size, with Cohen’s (1988, 1992) conventions applied to aid interpretation (0.01 small, 0.06 medium, 0.14 large).

Simple moderation analyses (in SPSS using PROCESS v4.0; Hayes, 2022) were used to test hypotheses that the predictive value of Time 1 sustained attention and social attention on child outcomes would differ by intervention approach. First, for each group, partial correlations were examined between Time 1 indicators of sustained/social attention and Time 2 V/NVDQ and VABS-ABC, controlling for Time 1 measures of the latter. Moderation analyses were then conducted where indicated (i.e., according to differential pattern of correlations—direction or strength of effect—in each group). Given multiple available indicators of social learning, those demonstrating strongest association with the given outcome were retained for inclusion in moderator analyses. Model parameters were estimated using ordinary least squares regression, with bootstrapped estimates and confidence intervals based on 5000 samples. Significant interactions were probed with the Johnson-Neyman approach to identify the range of values at which the moderator demonstrated a statistically significant effect at *p* < 0.05.

## Results

### Intervention Outcomes

**Fig. 1:**
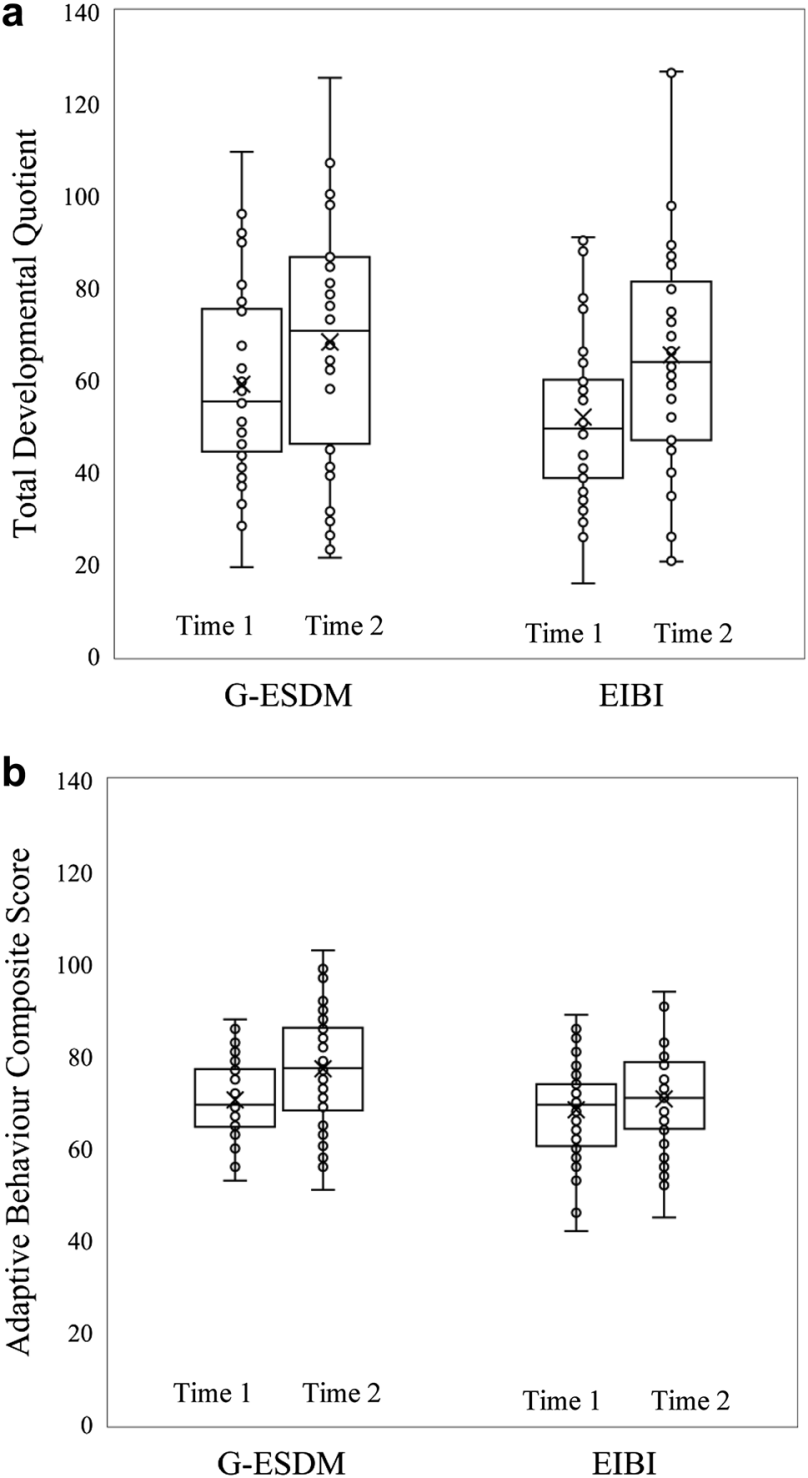
Change in standardized measures over one year of early intervention. box plots show the mean and distribution of (**a**) Developmental quotient and (**b**) Adaptive behaviour composite scores across group early start Denver model (G-ESDM) and early intensive behavioural intervention (EIBI) groups at time 1 and time 2. Scores are unadjusted for the group difference in child age at Time 1

Figure 1 presents the distribution of Time 1 and 2 MSEL-DQ and VABS-ABC scores for children receiving G-ESDM and EIBI. A general trend of increasing scores over time was evident, but with substantial within-group individual variation.

Table 2 shows adjusted means for MSEL-DQ, and VABS-ABC (controlling for group difference in child age, unadjusted scores are presented in supplementary materials). The ANCOVA on MSEL-DQ revealed a statistically significant main effect of Time [*F*(1,79) = 6.77, *p* = 0.011, partial *Ƞ*^*2*^ = 0.08], but not of Group [*F*(1,79) = 0.69, *p* = 0.793, partial *Ƞ*^*2*^ = 0.00], nor significant two-way interaction [*F*(1,79) = 2.79, *p* = 0.099, partial *Ƞ*^*2*^ = 0.03]. Similarly, the ANCOVA on VABS-ABC revealed a trend-level main effect of Time, [*F*(1,79) = 3.71, *p* = 0.058, partial *Ƞ*^*2*^ = 0.05], with no significant effect of Group, [*F*(1,79) = 0.32, *p* = 0.572, partial *Ƞ*^*2*^ = 0.00] or interaction [*F*(1,79) = 2.48, *p* = 0.119, partial *Ƞ*^*2*^ = 0.03].

**Table 2:** Measures of cognition and adaptive behaviour for the intervention groups at intake and follow-up—adjusted for chronological child age at intake

	G-ESDM *Madj*^*a*^*(SE)*		EIBI *Madj*^*a*^*(SE)*	
	T1	T2	T1	T2
Total DQ	57.90 (3.34)	65.66 (4.25)	52.92 (3.44)	67.69 (4.38)
VABS-ABC	69.41 (1.61)	75.67 (1.97)	69.61 (1.65)	72.51 (2.03)

*DQ*:Developmental quotient from mullen scales early learning, *VABS-ABC*:Vineland adaptive behaviour scales adaptive behaviour composite score

^a^ Adjusted for child age at intake

Follow-up ANCOVAs were conducted on MSEL domain AE scores (see supplementary materials, Table 5), indicating significant main effects of Time for visual reception, fine motor, receptive and expressive language AEs, and for summary VDQ but not NVDQ. Similar follow-up ANCOVAs on VABS domain scores revealed a significant main effect of Time for Communication, and trend-level effect for Socialisation, but no such effects for Motor or Daily Living Skills scores. No significant main effects of Group nor Group*Time interactions were evident here (supplementary materials, Table 5).

### Prognostic Indicators of Intervention Outcome

Table 3 shows the results of three hierarchical regressions testing Time 1 characterisation measures as prognostic indicators of outcomes for the cohort (and with relevant covariates identified based on correlations with the given outcome measure). In the model predicting Time 2 NVDQ, child sex and Time 1 ADOS-SA and NVDQ were each significant unique predictors entered together at Step 1, accounting for 65% of the variance. Eye-tracking metrics added at Step 2 contributed an additional 7% of variance, with sustained attention a significant unique predictor but ADOS-SA no longer so.

**Table 3:** Standardised regression coefficients from hierarchical regression models predicting non-verbal and verbal developmental quotient and adaptive behaviour composite scores

	Model 1 Non-Verbal DQ	Model 2 Verbal DQ	Model 3 Adaptive Behaviour
Step 1			
Child age at intake	–	–	− .28**
Sex (0 = Male 1 = Female)	− .21*	–	–
ADOS-SA	− .18*	− .23*	− .33**
ADOS-RRB	− .13	− .16*	− .06
T1 Non-verbal DQ	.72**	–	–
T1 Verbal DQ	–	.62**	–
T1 VABS-ABC	–	–	.57**
R^2^	.65	.69	.73
F	33.77**	58.50**	46.84**
Step 2			
Child age at intake	–	–	− .28**
Sex (0 = Male 1 = Female)	− .20*	–	–
ADOS-SA	− .06	− .12	− .25**
ADOS-RRB	− .09	− .11	− .02
T1 Non-verbal DQ	.64**	–	–
T1 Verbal DQ	–	.50**	–
T1 VABS-ABC	–		.48**
Sustained attention ^a^	.21*	.13	.13
Preferential social attention	-.04	.02	–
Joint attention – attention to target ^a^	− .06	.02	.07
Joint attention – first look to target	.07	–	–
Attention to playful adult	.15	.25**	.17*
Imitation performance ^a^	.08	–	–
R^2^	.72	.76	.78
F	16.89**	33.75**	25.10**
Δ R^2^	.07	.07	.05
Δ F	2.61*	5.37**	2.83*

^*^ < .05 ** < .001

^a^Sqrt transformed

*ADOS*:Autism diagnostic observation schedule, *SA*:Social affect total score *RRB*:Restricted repetitive behaviour total score, *DQ*:Developmental quotient score from mullen scales early learning, *VABS-ABC*:Vineland adaptive behaviour scales adaptive behaviour composite score

In the model predicting Time 2 VDQ, Time 1 ADOS-SA, ADOS-RRB and VDQ were significant predictors at Step 1, together accounting for 69% of the variance. Eye-tracking metrics entered at Step 2 contributed an additional 7% of variance, with attention to playful actor a significant unique predictor, but ADOS-SA again no longer so.

Finally, regarding Time 2 VABS-ABC, child age and Time 1 ADOS-SA and VABS-ABC carried significant value at Step 1, together accounting for 73% of variance. Attention to playful actor was a significant unique predictor when entered alongside other eye-tracking metrics at Step 2, together accounting for a further 5% of variance in VABS-ABC.

### Differential Predictors of Intervention Outcome

Partial correlations between Time 1 eye-tracking measures and Time 2 V/NVDQ and VABS-ABC (controlling for Time 1 levels of the same) revealed differential patterns of association across the groups (see Table 4). Within the G-ESDM group, higher Time 2 VDQ and NVDQ were associated with higher Time 1 sustained attention, preferential social attention, joint attention, imitation, and attention to playful actor, as well as male sex and lower Time 1 ADOS-SA. Within the EIBI group, higher Time 2 NVDQ was associated with lower Time 1 ADOS-SA and RRB, while higher Time 2 VDQ and VABS-ABC were both associated with higher Time 1 sustained attention, joint attention, and attention to a playful actor, and lower Time 1 ADOS-SA and RRB.

**Table 4:** Pearson’s partial correlations between baseline characteristics and outcome measures of cognition and adaptive behaviour (controlling for Time 1 measures) across intervention groups

	Total (N = 82)			G-ESDM (N = 42)			EIBI (N = 40)		
	NVDQ^b^	VDQ^c^	VABS-ABC^d^	NVDQ^b^	VDQ^c^	VABS-ABC^d^	NVDQ^b^	VDQ^c^	VABS-ABC^d^
Age at intake (months)	− .04	− .08	− .25*	.01	− .25	− .27	− .06	.00	.06
Sex (0 = Male 1 = Female)	− .32*	− .14	.04	− .37*	− .24	− .06	− .25	.05	.21
ADOS-SA	− .32*	− .36**	− .24*	− .29	− .32*	− .20	− .34*	− .43*	− .39*
ADOS-RRB	− .25*	− .29*	− .14	− .16	− .13	− .00	− .40*	− .57**	− .51**
Sustained Attention ^a^	.34*	.32*	.20	.58**	.32*	.08	.04	.33*	.39*
Preferential social attention	.14	.26*	.17	.45*	.44*	.16	-.20	.05	.27
Joint attention – attention to target ^a^	.26*	.35*	.20	.42*	.33*	.03	.06	.33*	.44*
Joint attention—first look	.26*	.20	.13	.31	.15	.01	.21	.26	.36*
Playful actor	.34*	.51**	.35	.43*	.57**	.31*	.21	.41*	.42*
Imitation score ^a^	.25*	.12	− .03	.33*	.24	− .02	.18	− .01	.15

^*^ < .05 ** < .001

^a^Sqrt Transformed ^b^Controlling for time 1 NVDQ ^c^ Controlling for time 1 VDQ ^d^ Controlling for time 1 Adaptive behaviour

*ADOS*:Autism diagnostic observation schedule, *SA*:Social Affect total score *RRB*: Restricted repetitive behaviour total score, *V/NV DQ*:Verbal/Non-verbal developmental quotient score from mullen scales early learning, *VABS-ABC*: Vineland adaptive behaviour scales adaptive behaviour composite score

**Fig. 2:**
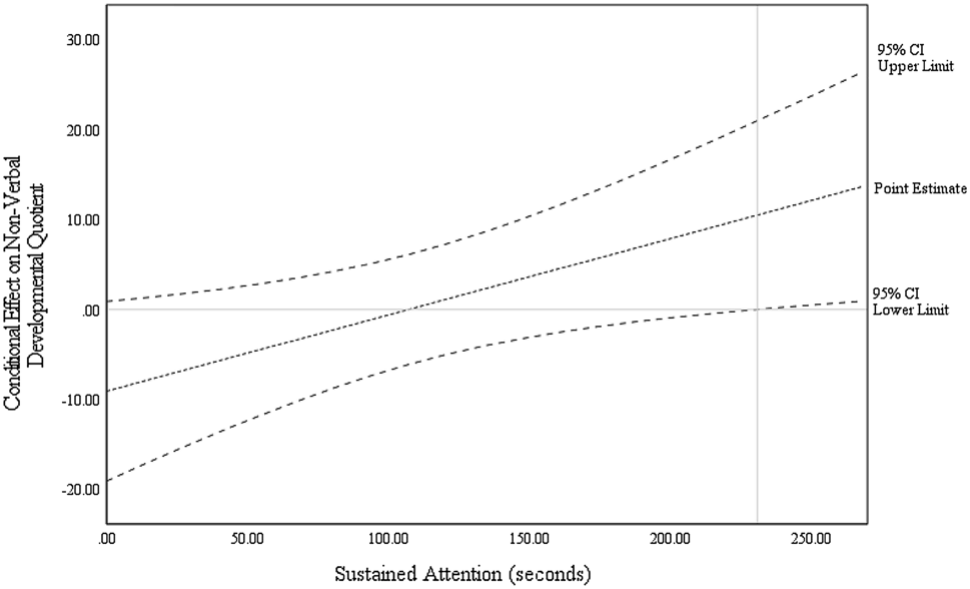
Conditional effects of sustained attention on non-verbal developmental quotient among children receiving early intensive behaviour intervention (EIBI) or group early start denver model (G-ESDM)

Moderation analyses were conducted to determine any moderating effect of intervention Group on the association of Time 1 sustained and social attention on the various outcome measures (see Table 5). Model 1 predicting Time 2 NVDQ included a significant Group*sustained attention interaction [*F*(1,73) = 4.09, *p* = 0.047]. Controlling for relevant covariates, conditional effects indicated that within the G-ESDM group, children with higher Time 1 sustained attention had higher Time 2 NVDQ [*b* = 1.43, 95%CI(0.57–2.30), *p* = 0.002] with no such predictive value of Time 1 sustained attention for Time 2 NVDQ in the EIBI group [*b* = 0.21, 95%CI(− 0.73–1.16), *p* = 0.655]. The identified region of significance (Fig. 2) suggested that children in the G-ESDM group with sustained attention > 230 (out possible total 300) seconds had higher Time 2 NVDQ.

**Table 5:** Regression analyses examining the moderation effect of group on the relationship between indicators of sustained and social attention, and child outcome measures after 1 year of intervention

	b	SE	t	p	R^2^
Model 1. Sustained attention on time 2 non-verbal DQ					.69
Constant	39.66	11.28	3.62	.001	
Sustained attention (mean centred)	0.20	0.50	0.45	.655	
Group (EIBI = 1, G-ESDM = 0)	− 0.06	2.67	0.01	.995	
Group * sustained attention	1.24	0.60	2.02	.047	
T1 Non-verbal DQ (covariate)	0.79	0.10	7.75	< .001	
Child sex (Female = 1, Male = 0) (covariate)	− 10.38	3.79	− 2.66	.010	
T1 ADOS-SA (covariate)	− 0.77	0.52	− 1.65	.103	
T1 ADOS-RRB (covariate)	− 1.63	0.79	− 1.86	.067	
Model 2. Preferential social attention on time 2 non-verbal DQ					.66
Constant	41.75	11.15	3.52	.001	
Preferential social attention (mean centred)	− 0.41	1.08	− 0.47	.638	
Group (EIBI = 1, G-ESDM = 0)	0.53	2.94	0.16	.876	
Group * Preferential social attention	2.53	1.76	1.64	.106	
T1 Non-verbal DQ (covariate)	0.81	0.11	7.41	< .001	
Child sex (Female = 1, Male = 0) (covariate)	− 9.14	4.33	− 2.13	.037	
T1 ADOS-SA (covariate)	− 0.93	0.51	− 1.93	.058	
T1 ADOS-RRB (covariate)	− 1.87	0.85	− 1.96	.054	
Model 3. Preferential social attention on time 2 verbal DQ					.72
Constant	62.77	9.85	5.82	< .001	
Preferential social attention (mean centred)	0.79	1.06	0.69	.491	
Group (EIBI = 1, G-ESDM = 0)	3.76	3.09	1.01	.314	
Group * preferential social attention	2.85	1.84	1.69	.094	
T1 Verbal DQ (covariate)	0.62	0.55	-2.63	< .001	
T1 ADOS-SA (covariate)	− 1.44	1.01	− 2.47	.010	
T1 ADOS-RRB (covariate)	− 2.57	0.08	7.02	.016	
Model 4. Sustained attention on time 2 adaptive behaviour					.75
Constant	54.27	10.64	5.24	< .001	
Sustained attention (mean centred)	0.38	0.29	1.55	.126	
Group (EIBI = 1, G-ESDM = 0)	2.43	1.93	1.43	.157	
Group * sustained attention	0.01	0.31	0.16	.988	
T1 adaptive behaviour (covariate)	0.61	0.11	6.04	< .001	
Child age at intake	− 2.47	0.11	− 2.66	.010	
Child sex (Female = 1, Male = 0) (covariate)	0.13	1.79	0.06	.952	
T1 ADOS-SA (covariate)	− 0.86	0.24	− 3.94	.000	
T1 ADOS-RRB	− 0.71	0.46	− 1.63	.108	
Model 5. joint attention on time 2 adaptive behaviour					.75
Constant	50.40	10.57	5.12	< .001	
Response to Joint Attention—attention to target (mean centred)	4.69	2.76	1.52	.133	
Group (EIBI = 1, G-ESDM = 0)	2.41	1.88	1.46	.148	
Group * response to joint attention	0.86	3.57	0.14	.892	
T1 adaptive behaviour (covariate)	0.64	0.11	6.90	< .001	
Child age at intake	− 0.23	0.10	-2.50	.015	
Child sex (Female = 1, Male = 0) (covariate)	0.53	1.69	0.29	.769	
T1 ADOS-SA (covariate)	− 0.84	0.25	− 3.86	< .001	
T1 ADOS-RRB (covariate)	− 0.63	0.49	− 1.35	.181

Moderator models testing Group*preferential social attention interactions, were non-significant, for either Time 2 NVDQ [Model 2: *F*(1,73) = 2.68, *p* = 0.106] and Time 2 VDQ outcomes [Model 3: *F*(1,74) = 2.87, *p* = 0.094], as were those for Time 2 VABS-ABC outcome testing Group*sustained attention [Model 4: *F*(1,72) = 0.00, *p* = 0.988] and Group*response to joint attention interactions [Model 5: *F*(1,72) = 0.02, *p* = 0.892].

## Discussion

We examined outcomes, and the predictors thereof, of matched groups of autistic preschool-aged children receiving either G-ESDM or EIBI within the same community. Our prediction that children in both groups would make gains, at group mean-level, on standardized measures was supported, with an increase in average scores over time evident in both groups across measures of development and adaptive behaviour. A significant increase in skills was evident across all MSEL-AE subscales. The standard scores indicated significant change in overall DQ and this appeared to be driven predominately by an increase in VDQ (with no significant change in NVDQ scores for either group). A trend-level change in adaptive behaviour was also evident, with further examination of the VABS subscales indicating a significant increase in Communication skills (but not other subscale scores) for children in either group. This finding is aligned with and extends on previous recent research suggesting that, on average, children benefit from both ESDM and EIBI, delivered in both group and 1:1 settings, with no clear evidence of superiority of one program over another (Rogers et al., 2019, 2021; Vivanti et al., 2019).

Parents of autistic children have described the process of identifying, and selecting an appropriate program of support for their child as frustrating and stressful (Bent et al., 2022; Wilson et al., 2018). The lack of group-level differences found here suggests that key common elements across effective interventions may be more important that the specific “brand name” of the program. Both EIBI and G-ESDM intervention approaches deliver support consistent with local good practice guidelines current at the time of the study (Prior & Roberts, 2012; Roberts & Williams, 2016), which recommend that autistic children receive 15–25 h of intervention per week, at a ratio from 1:1 to 1:4. Both approaches are also based on behavioural learning principles, employ a research-informed manualised curriculum, are delivered by interventionists with formal training, develop individualised child learning goals in consultation with families, monitor progress against these goals and adapt accordingly. These similarities may be more impactful on child learning outcomes than the differences between approaches.

Individual differences in child outcomes were evident within each group, with many children making gains across one year of early intervention, and others demonstrating limited change. We sought to understand which baseline child characteristics might be associated with the degree of individual response to intervention—whether prognostically (i.e., irrespective of intervention received) or predictively (i.e., specifically in the context of one or other approach). Our hypothesis that core autism features, developmental level, domain-general attention skills (e.g., sustained attention) and social-domain specific skills (i.e., social interest, joint attention, imitation) would be prognostically associated with outcomes was partially supported. Baseline sustained attention was associated with outcome NVDQ, over and above the predictive stability of this same measure, the predictive value of early ADOS-SA and RRB scores, and indicators of specific social attention. Sustained attention may therefore be a more influential prognostic indicator of non-verbal developmental skills than core autism features or indicators of social attention. This is consistent with the notion that a child’s capacity to regulate and maintain attention is a key determinant of the degree to which they can maximise learning opportunities (Fisher, 2019).

A key objective of this study was to determine any specific predictive relationship between sustained attention and outcomes, by intervention approach. Indeed, we found higher sustained attention to be associated with NVDQ at outcome specifically for the G-ESDM group (and not for children in EIBI). However, this finding was contrary to our prediction that domain-general skills might more strongly predict outcomes for children receiving EIBI. Plausibly, sustained attention skills may support child outcomes through naturalistic G-ESDM, allowing a child to capitalise on available learning opportunities within a relatively unstructured environment. That is, sustained attention may be *less* critical for child outcomes within EIBI where this capacity may be directly targeted as an intervention goal early in the learning process (i.e., “learning how to learn”) such that the EIBI approach and environment means learning is less reliant on a child’s intrinsic sustained attention capacity, than is true for G-ESDM.

We similarly examined the prognostic and predictive influence of social attention on intervention outcomes. Among the various indicators of social attention, attention to playful adult contributed significant unique variance to predicting verbal skills and adaptive behaviour, for children receiving G-ESDM and EIBI. This is consistent with past research implicating observational social learning for language acquisition (Kuhl, 2007) and suggests that *social interest* may be a more influential prognostic indicator than metrics such as joint attention which require more direct eye contact. Our hypothesis that social attention would be more strongly predictive of outcomes for children receiving G-ESDM was partially supported in that social attention indicators—preferential social attention and attention to playful adult—were correlated with V/NVDQ outcomes in the G-ESDM (and not EIBI) group. However, there was no evidence of moderation by Group after the inclusion of relevant covariates, suggesting that the association between social attention and learning outcomes does not differ by intervention approach.

A key limitation of this study was the non-randomised design with selection biases and unmeasured/controlled factors potentially underscoring the observed effects. To mitigate the potential impact of sample characterisation differences, we sought to match the groups on baseline developmental levels, verbal abilities, adaptive behaviour and core autism features, and we statistically controlled for enduring differences in child age. Furthermore, conduct within community-based (rather than highly controlled) intervention settings means this research may reflect a more representative and diverse sample than traditionally true for childhood autism research (Maye et al., 2022; Pellecchia et al., 2018) with cohort heterogeneity—in child characteristics and intervention response—a strength of this study. However, future research should include a broader range of socio-demographic characterisation measures relating to children, families, and service providers – to aid our understanding of the generalisability of research findings.

Measures of intervention fidelity were utilised by service providers for training and coaching purposes, to ensure quality of service delivery, however this data was not systematically collected for research purposes as part of the study design. Future studies incorporating a high degree of methodological rigour, including fidelity measures, and blinded outcome measures double-coded for inter-rater reliability would increase certainty in the study findings. Further consideration of factors known to influence the uptake and implementation of interventions including the cost, training requirements, and stakeholder perceptions of interventions should also be considered.

Related studies have examined parents’ perspectives of their child’s participation in group-based NDBI, reporting largely positive experiences including a strong allegiance towards the staff and intervention approach—albeit with difficulties transitioning away from a time-limited program (Bent et al., 2022). Future studies, particularly those examining intervention outcomes, should similarly seek to incorporate measures of social validity, given changing community perceptions, and the importance of engaging with stakeholders as equal partners.

While our cohort sample size was adequate, it was small when the groups were treated separately, especially following the necessary exclusion of participants to achieve group matching, and future research would benefit from larger samples for well-powered tests of differential within-group predictors. While developmental skills and adaptive behaviours are important indicators of later-life outcomes and disability, future studies should consider a broader range of proximal outcomes in early childhood, such as quality of life, peer relationships and community participation.

Few studies have directly compared the outcomes of autistic children receiving different intervention approaches, and limited evidence is available to support parents and service providers decision-making. We found no evidence for superiority of one program over the other, with substantial outcome variability evident for children enrolled in both the EIBI and G-ESDM programs. Attention to playful adult was prognostic of verbal and adaptive behaviour outcomes in this cohort, but it remains unclear if this effect would apply similarly to other intervention approaches or indeed to the ‘natural course’ of autism. We also identified sustained attention as a differential predictor of non-verbal outcomes, with children with higher sustained attention making more gains in G-ESDM (but with no such association in the context of EIBI). Given these findings, we suggest that clinicians continue to partner with families to make decisions on the style and intensity of supports that may suit an individual child using an appropriate evidence-based framework (Trembath et al., 2021). While the question of “what works for whom?” remains open, our findings suggest that fine-grained measurement of learning skills offers promise towards the selection of intervention approaches that might best meet individual child learning needs.

### Supplementary Information

Below is the link to the electronic supplementary material.

Supplementary file1 (DOCX 31 KB)
