# Supplementary material for: Predictors of Developmental and Adaptive Behaviour Outcomes in Response to Early Intensive Behavioural Intervention and the Early Start Denver Model
Source: J Autism Dev Disord. 2023 May 12;54(7):2668–81. doi: 10.1007/s10803-023-05993-w (PMC11286629; doi:10.1007/s10803-023-05993-w)
Supplement: Supplementary file 1 — Supplementary file1 (DOCX 31 KB) [file 10803_2023_5993_MOESM1_ESM.docx]

**Supplementary Material**

Pearson’s partial correlation analyses between eye tracking metrics and standardised measures of social communication skills and difficulties - supporting the concurrent validity of eye tracking metrics are presented in Table 1 and 2, respectively.

**Table 1.**

Concurrent associations between eye-tracking measures

|  | Preferential social attention | Joint attention – duration of attention to target ^a^ | Joint attention - proportion of first looks to target | Attention to a playful adult | Imitation performance score ^a^ |
| --- | --- | --- | --- | --- | --- |
| Sustained attention ^a^ | .480** | .680** | .457** | .385** | .264* |
| Preferential social attention |  | .544** | .392** | .508** | .251* |
| Joint attention – duration of attention to target ^a^ |  |  | .644** | .470** | .205 |
| Joint attention - proportion of first looks to target |  |  |  | .337** | .195 |
| Attention to a playful adult |  |  |  |  | .342* |

*Note.* *<.05 **<.001 ^a^ Sqrt Transformed

**Table 2.**

Concurrent associations between eye-tracking measures and standard measures at intake to intervention (Time 1)

|  | ADOS SA | VABS Communication SS | VABS Social SS |
| --- | --- | --- | --- |
| Sustained attention ^a^ | -.450** | .599** | .437** |
| Preferential social attention | -.324* | .398** | .336** |
| Joint attention – duration of attention to target ^a^ | -.435** | .499** | .350** |
| Joint attention - proportion of first looks to target | -.214* | .302* | .156 |
| Attention to a playful adult | -.509* | .386** | .245* |
| Imitation performance score ^a^ | -.356** | .188 | .200 |

*Note.* *<.05 **<.001 ^c^ ADOS = Autism Diagnostic Observation Schedule. SA = Social Affect Total Score. VABS = Vineland Adaptive Behaviour Scales. SS = Standard Score

Sample characteristics before and after imputation of missing data using Expectation Maximization is presented in Table 3.

**Table 3.**

*Sample Characteristics before and after Imputation of Missing Data*

|  | Original | | Imputed | |
| --- | --- | --- | --- | --- |
|  | N | M(SD) | N | M(SD) |
| Child Sex, (Male, N %) | 82 | 65 (79.2% females) | 82 |  |
| Age at Intake (months) | 82 | 37.20 (10.03) | 82 |  |
| Intervention Days/week | 82 | 3.59 (0.61) | 82 |  |
| ADOS-2 SA | 77 | 13.09 (3.85) | 82 | 13.05 (3.75) |
| ADOS-2 RRB | 77 | 5.00 (1.83) | 82 | 4.97 (1.78) |
| ADOS-2 CSS | 77 | 7.03 (1.91) | 82 | 7.02 (1.85) |
| T1 Non Verbal DQ | 80 | 64.55 (18.17) | 82 | 64.21 (18.26) |
| T1 Verbal DQ | 81 | 48.24 (25.10) | 82 | 48.29 (24.95) |
| T1 Adaptive Behaviour | 80 | 69.53 (9.56) | 82 | 69.51 (9.46) |
| T2 Non Verbal DQ | 78 | 70.49 (22.38) | 82 | 70.29 (22.42) |
| T2 Verbal DQ | 78 | 62.86 (27.44) | 82 | 62.49 (27.49) |
| T2 Adaptive Behaviour | 73 | 74.19 (12.24) | 82 | 74.13 (12.05) |
| Valid N (listwise) | 67 |  | 82 |  |

*Note.* Statistics are mean (standard deviation), unless otherwise specified, *p* values derived from chi square and simple t-tests. ADOS = Autism Diagnostic Observation Schedule. SA = Social Affect Total Score RRB = Restricted Repetitive Behaviour Total Score. DQ = Developmental Quotient Score from Mullen Scales Early Learning. Adaptive Behaviour = Adaptive Behaviour Composite Score from Vineland Adaptive Behaviour Scales.

**Table 4.**

*Measures of Cognition and Adaptive Behaviour for the Intervention Groups at Intake and Follow-up (Unadjusted for Chronological Child Age at Intake)*

|  | G-ESDM (*n* = 42) *M(SE)* | | EIBI (*n* = 40) *M(SE)* | |
| --- | --- | --- | --- | --- |
|  | T1 | T2 | T1 | T2 |
| Visual Reception AE | 20.34 (0.97) | 30.52 (1.63) | 26.28 (1.70) | 38.57 (2.33) |
| Fine Motor AE | 21.12 (1.00) | 28.57 (1.46) | 27.03 (1.72) | 36.93 (2.10) |
| Receptive Language AE | 15.36 (1.50) | 26.57 (1.86) | 18.83 (1.98) | 33.77 (2.58) |
| Expressive Language AE | 16.81 (1.33) | 26.88 (1.90) | 19.66 (1.86) | 31.61 (2.21) |
| Non-Verbal DQ | 66.95(2.80) | 71.35 (3.46) | 61.32 (2.87) | 69.18 (3.58) |
| Verbal DQ | 51.87 (3.98) | 65.00 (4.51) | 44.54 (3.76) | 59.85 (4.06) |
| Total DQ | 59.07 (3.21) | 68.28 (3.95) | 51.69 (2.82) | 64.94 (3.84) |
|  |  |  |  |  |
| Communication SS | 71.71 (1.92) | 80.52 (2.42) | 67.67(2.72) | 71.96 (2.73) |
| Daily Living SS | 76.13 (1.50) | 79.12 (2.21) | 71.27(1.74) | 71.98 (1.77) |
| Socialisation SS | 70.34 (0.93) | 75.38 (1.68) | 68.54 (1.87) | 69.65 (1.60) |
| Motor Skills SS | 79.85 (1.20) | 81.14 (1.69) | 80.05 (2.10) | 78.09 (1.63) |
| Vineland ABC | 70.57 (1.25) | 77.32 (1.91) | 68.40 (1.69) | 70.78 (1.72) |

Results of follow-up ANCOVAs on MSEL and VABS subdomain scores are presented in Table 4.
 **Table 5**

*Measures of Development and Adaptive Behaviour for the Intervention Groups at Intake and Follow-up, Adjusted for Chronological Child Age at Intake*

|  | G-ESDM  *Madj^a^(SE)* | | EIBI  *Madj^a^(SE)* | | Time effect | | Group effect | | Interaction | |
| --- | --- | --- | --- | --- | --- | --- | --- | --- | --- | --- |
|  | T1 | T2 | T1 | T2 | *p* | partial η2 | *p* | partial η2 | *p* | partial η2 |
| Visual Reception AE ^b^ | 22.46 (1.42) | 33.39 (2.12) | 24.11 (1.44) | 36.10 (2.15) | .002 | .11 | .501 | .01 | .592 | 00 |
| Fine Motor AE ^b^ | 23.93 (1.35) | 31.14 (1.87) | 24.08 (1.39) | 34.23 (1.92) | .001 | .12 | .507 | .01 | .127 | 03 |
| Receptive Language AE ^b^ | 17.50 (1.85) | 29.09 (2.38) | 16.58 (1.90) | 31.13 (2.45) | <.001 | .13 | .998 | .00 | .311 | .01 |
| Expressive Language AE ^b^ | 19.30 (1.66) | 29.28 (2.18) | 17.04 (1.70) | 29.10 (2.25) | <.001 | .14 | .622 | .00 | .166 | .02 |
| Non-Verbal DQ | 64.21 (3.05) | 68.58 (3.82) | 64.21 (3.14) | 72.09 (3.94) | .423 | .01 | .727 | .00 | .393 | .01 |
| Verbal DQ | 51.11 (4.28) | 62.94 (4.72) | 45.33 (4.41) | 62.01 (4.86) | .012 | .08 | .617 | .00 | .301 | .01 |
| Communication SS | 71.99 (2.58) | 78.29 (2.79) | 67.38 (2.65) | 74.30 (2.87) | <.001 | .16 | .274 | .02 | .844 | .00 |
| Daily Living SS | 75.22 (1.77) | 77.82 (2.20) | 72.22 (1.82) | 73.35 (2.27) | .379 | .01 | .193 | .02 | .579 | .00 |
| Socialisation SS | 69.18 (1.58) | 73.69 (1.76) | 69.75 (1.62) | 71.42 (1.81) | .082 | .04 | .727 | .00 | .155 | .03 |
| Motor Skills SS | 77.95 (1.81) | 78.94 (1.75) | 82.05 (1.86) | 80.40 (1.80) | .755 | .00 | .255 | .02 | .345 | .01 |

*Note.* AE = Age Equivalent scores from Mullen Scales Early Learning; DQ = Developmental Quotient from Mullen Scales Early Learning; ABC = Adaptive Behaviour Composite Score from Vineland Adaptive Behaviour Scales ^a^ Adjusted for child age at intake. ^b^ Data was square root transformed for analysis, means for untransformed data are included for interpretability.
